# Effect of a teaching intervention based on planned behavior theory on female nurses' preventive behaviors towards the osteoporosis. A quasi-experimental study or a randomized controlled trial

**DOI:** 10.17533/udea.iee.v44n1e11

**Published:** 2026-03-30

**Authors:** Ali Dehghani, Farzaneh Afrough Jahromi, Zohreh Badiyepeymaiejahromi

**Affiliations:** 2 M.Sc. Email: fafrough2020@gmail.com https://orcid.org/0000-0001-5237-9102 fafrough2020@gmail.com; 3 Ph.D. Associate professor. Email: z.badiyepeyma@gmail.com. https://orcid.org/0000-0001-6643-036X z.badiyepeyma@gmail.com; 4 Department of Community Health Nursing, School of Nursing, Jahrom University of Medical Sciences, Jahrom, Iran. Department of Community Health Nursing School of Nursing Jahrom University of Medical Sciences Jahrom Iran; 5 Department of Nursing, School of Nursing and Midwifery, Shiraz University of Medical Sciences, Shiraz, Iran. Department of Nursing School of Nursing and Midwifery Shiraz University of Medical Sciences Shiraz Iran

**Keywords:** osteoporosis, health behavior, education, nursing, theory of planned behavior, nurses., osteoporosis, conductas relacionadas con la salud, educación en enfermería, teoría del comportamiento planificado, enfermeras y enfermeros, osteoporose, comportamentos relacionados com a saúde, educação em enfermagem, teoria do comportamento planejado, enfermeiras e enfermeiros

## Abstract

**Objective.:**

Examine the effect of a teaching intervention based on planned behavior theory on female nurses' preventive behaviors toward osteoporosis.

**Methods.:**

In this quasi-experimental study, 100 female nurses working at two hospitals affiliated with Jahrom University of Medical Sciences in southern Iran were place in the intervention and control groups. Data were collected using a questionnaire that included demographic information and constructs of planned behavior theory. Data were collected in three phases: pretest, posttest one month after the teaching intervention, and posttest two months after the teaching intervention. Four sessions of 45-60 min training program were held within the period of a month for the experimental group at classrooms equipped with a video projector and whiteboard. No intervention was given to the control group.

**Results.:**

The findings showed that the intervention and control groups were similar in terms of demographic variables before the intervention. There were significant differences in the means of attitude, abstract norms, behavioral intent, and preventive behavior in the intervention group compared to the control group one and two months after the teaching intervention (*p*<0.05). However, the mean perceived control of behavior did not differ significantly between the intervention and control groups (*p*=0.15). In the control group, the means of attitude, abstract norms, behavioral intent, and behavior did not differ significantly regarding osteoporosis prevention one and two months after the intervention compared to before the teaching intervention (*p*>0.05). The intervention had small effects on attitudes (Partial eta squared: η²p = 0.025 to 0.047), moderate effects on perceived behavioral control (η²p = 0.038 to0.054) and behavioral intention (η²p = 0.116 to 0.126), large effects on abstract norms (η²p = 0.282 to 0.295), and very large effects on behavior (η²p = 0.365 to 0.498).

**Conclusion.:**

According to the results of this study, planned behavior theory may influence the preventive behavior of osteoporosis in female nurses. Therefore, this theory could serve as a framework for designing and conducting teaching interventions to prevent osteoporosis and promote health in women.

## Resumen

**Objetivo.:**

Examinar el efecto de una intervención docente basada en la teoría del comportamiento planificado sobre los comportamientos preventivos de las enfermeras frente a la osteoporosis.

**Métodos.:**

En este estudio cuasi-experimental, 100 enfermeras que trabajaban en dos hospitales afiliados a la Universidad de Ciencias Médicas de Jahrom (sur de Irán), fueron asignadas aleatoriamente al grupo de intervención o al grupo de control. La intervención educativa consistió en cuatro sesiones de un programa de entrenamiento de 45-60 minutos de duración durante un mes para el grupo experimental. No se realizó ninguna intervención con el grupo de control. Los datos se recopilaron mediante un cuestionario que incluía información demográfica y preguntas sobre los constructos de la teoría del comportamiento planificado. Los datos se recopilaron en tres fases: basal, un mes y dos meses después de la intervención educativa.

**Resultados.:**

Los hallazgos mostraron que los grupos de intervención y control eran similares en términos de variables demográficas antes de la intervención. Se observaron diferencias significativas en las medias de actitud, normas abstractas, intención conductual y comportamiento preventivo en el grupo de intervención en comparación con el grupo de control uno y dos meses después de la intervención educativa (*p*<0.05). Sin embargo, la media del control percibido del comportamiento no difirió significativamente entre los dos grupos de studio (*p*=0.15). En el grupo de control, las medias de actitud, normas abstractas, intención de comportamiento y comportamiento no difirieron significativamente en cuanto a la prevención de la osteoporosis uno y dos meses después de la intervención (p>0.05). La intervención tuvo efectos pequeños en las actitudes (Partial eta squared: η²p =0.025 a 0.047), efectos moderados en el control conductual percibido (η²p=0.038 a 0.054) y en la intención conductual (η²p=0.116 a 0.126), efectos grandes en las normas abstractas (η²p=0.282 a 0.295), y efectos muy grandes en el comportamiento (η²p=0.365 a 0.498).

**Conclusión.:**

Según los resultados de este estudio, la teoría del comportamiento planificado puede influir en el comportamiento preventivo ante la osteoporosis en las enfermeras. Por lo tanto, esta teoría podría servir como marco para diseñar y llevar a cabo intervenciones docentes para prevenir la osteoporosis y promover la salud en las mujeres.

## Resumo

**Objetivo.:**

Examinar o efeito de uma intervenção pedagógica baseada na teoria do comportamento planejado nos comportamentos preventivos de enfermeiras em relação à osteoporose.

**Métodos.:**

Neste estudo quase experimental, 100 enfermeiras que trabalham em dois hospitais afiliados à Universidade de Ciências Médicas de Jahrom, no sul do Irã, foram colocadas nos grupos de intervenção e controle. Os dados foram coletados por meio de um questionário que incluía informações demográficas e conceitos da teoria do comportamento planejado. Os dados foram coletados em três fases: pré-teste, pós-teste um mês após a intervenção de ensino e pós-teste dois meses após a intervenção de ensino. Quatro sessões de um programa de treinamento de 45 a 60 minutos foram realizadas no período de um mês para o grupo experimental em salas de aula equipadas com um projetor de vídeo e quadro branco. Nenhuma intervenção foi realizada no grupo controle.

**Resultados.:**

Os resultados mostraram que os grupos de intervenção e controle eram semelhantes em termos de variáveis demográficas antes da intervenção. Houve diferenças significativas nas médias de atitude, normas abstratas, intenção comportamental e comportamento preventivo no grupo de intervenção em comparação com o grupo controle um e dois meses após a intervenção de ensino (*p*<0.05). No entanto, a média do controle percebido do comportamento não diferiu significativamente entre os grupos de intervenção e controle (*p*=0.15). No grupo controle, as médias de atitude, normas abstratas, intenção comportamental e comportamento não diferiram significativamente em relação à prevenção da osteoporose um e dois meses após a intervenção em comparação com antes da intervenção de ensino (*p*>0.05). A intervenção teve pequenos efeitos nas atitudes (eta quadrado parcial: η²p=0.025 a 0.047), efeitos moderados no controle comportamental percebido (η²p=0.038 a 0.054) e na intenção comportamental (η²p=0.116 a 0.126), efeitos grandes nas normas abstratas (η²p=0.282 a 0.295) e efeitos muito grandes no comportamento (η²p=0.365 a 0.498).

**Conclusão.:**

De acordo com os resultados deste estudo, a teoria do comportamento planejado pode influenciar o comportamento preventivo da osteoporose em enfermeiras. Portanto, essa teoria poderia servir como uma estrutura para projetar e conduzir intervenções de ensino para prevenir a osteoporose e promover a saúde nas mulheres.

## Introduction

Osteoporosis is a systemic disease. It causes decreased bone mass and bone destruction.^1^ This disease is the most common among bone metabolic diseases. It results in bone fragility.^2^ Osteoporosis is defined by reduced height, kyphosis, fractures, and respiratory disorders in older age groups.^3^ Osteoporosis is affected by various factors, including environmental, genetic, and lifestyle factors.^4^ It is clear that as life expectancy and life span increase, so does the prevalence of osteoporosis and related fractures.^5^ Women are undeniably exposed to osteoporosis eight times more than men.^6^ The number of osteoporosis-related fractures in the European Union is projected to rise from 4.3 million in 2019 to 5.3 million in 2034.^1^ Osteoporosis affects 10-30% of women over 40 years old and 10% of men in seven developed countries in Asia and Oceania.^5^ A study in Iran reported the following: 32% of women had osteoporosis, 51% had low bone density, and 32% had osteoporosis in the lumbar region, 21% in the vertebrae, 21% in the neck of the femur, and 21% in the hip joint.^7^ According to a systematic review in Iran, prevalence of osteoporosis has been reported 38% in women and 25% in men.^8^ Osteoporosis has significant effects on psychosocial functions including anxiety, depression, low independence in activity of daily living, and low self-esteem. Preventing this complication is important.^9^^,^^10^ Various occupations affect health differently. Nursing is recognized as one of the high risk occupation with physical, psychological, and social tensions.^11^ Nursing is inseparable of healthcare and includes health promotion, disease prevention and care of patients with physical and psychological disorders. Nurses have responsibility to response to problems in patients, people, families and groups.^12^

Nurses’ health is at risk more than the general population. The World Health Organization in a study on stressful occupations reported that out of 130 occupations, nurses rank 27 in terms of health problems.^4^ Studies have shown that nurses suffer from diseases such as musculoskeletal, cardiovascular, respiratory, infectious, and psychological diseases. These diseases are more common in female nurses than male ones.^13^ Night shift could be the main factor for decreasing vitamin D resulting osteoporosis as they work in an environment without sunshine. Prospective studies have shown that working night shift more than 20 years is associated with increasing risk of hip and wrist factures.^14^^,^^15^ Preventive strategies of osteoporosis include increasing bone density and minimizing trend of bone decreasing through health education and promotion.^16^ Osteoporosis is preventable, as it is latent and not diagnosed until late stage.^3^ Health education is central for any health activity. It is fundamental for health promotion.^17^ Value and effectiveness of health education plans depend on proper using of theories.^18^ Theories help health education planners to think beyond personal issues during need assessment and developing plan.^19^ One of the related theories is planned behavior theory which could be effective in predicting health behaviors. According to this theory, the most important predictor of behavior management is intent for behavior, which consists of motivational factors affecting behavior. It is directed by three factors of 1. Attitude to behavior 2. Mental norms 3. Perceived control of behavior. These three factors predict intent of behavior.^20^ Attitude reflects positive or negative evaluation of behavior. Mental norms refer to perceived pressure for doing or not doing. Perceived control of behavior is perception about easy or difficulty of performing behavior. Behavior intent indicates severity of will for performing behavior.^21^

This theory has been employed in predicting and explaining various spectrum of behavior including diet behavior in students and adults,^16^^,^^22^ physical activity,^23^ banks,^24^ construct industry,^25^ preventive behavior in chronic conditions.^26^^,^^27^ Studies showed that planned behavior theory result in maternal care, encouraging normal vaginal delivery.^26^^,^^28^ This theory also results in improving osteoporosis behaviors in studies in female teachers ^29^ and on patients with type 2 diabetes.^27^ Chronic conditions and osteoporosis are increasing in women, particularly nurses. Osteoporosis is preventable, and health education through planned behavior theory could predict and change health behavior. This study was conducted to examine the effect of teaching intervention based on planned behavior on preventive behaviors of osteoporosis in female nurses.

## Methods

Type of study and sample. This is a quasi-experimental study, which was conducted on female nurses working at two Motahari and Seyed-shohada hospitals affiliated to Jahrom University of Medial Sciences (South of Iran) in month of 2024 to month of 2025. Sample size was calculated 50 in each group and 100 in total based on Altman Normogram^30^ considering Standard difference= 0.85, confidence interval 95% and power 90%.^31^

Inclusion criteria were female nurses aged 30 to 59 years, having at least one-year clinical working experience, not taking part in other experimental studies on osteoporosis, not taking part in workshop or teaching class about osteoporosis over past six months, not taking corticosteroid or other medications affecting osteoporosis, and not having osteoporosis. Exclusion criteria were not attending in the teaching sessions, being absentee more than two sessions, and not having willingness to continue the study. For recruitment, we obtained a list of female nurses from the nursing offices at Seyed-shohada (interventions group) and Motahari (control group) hospitals. Those who were eligible were then extracted from the primary list. Finally, 50 nurses from each hospital were selected using simple random sampling. The control group was selected from another hospital to minimize contamination bias. 

Data Collection Tools. In this study, data were collected using demographic and constructs of planned behavior theory questionnaires (attitude, abstract norms, perceived control of behavior, behavioral intent, and behavior). Data collection tool includes six demographic and background questions and a questionnaire consisting five parts: (i) *attitude measurement*: 15 questions, range of score 15 - 75 ; (ii) *mental norms measurement*: 8 questions, range of score 8 - 40; (iii) *perceived control of behavior measurement*: 6 questions, range of score 6 - 30; (iv) behavioral intent measurement: 8 questions, range of score 8 - 40; and (v) 8 questions related to *behavior* as once per day, twice or more per day, once per week, twice or more per week, and never with a range of score 8 - 40. Higher score in all dimensions indicates better behavior. Validity and reliability of this questionnaire have been reported in a study by Jalili *et al*.^32^ For psychometrics of the questionnaire in Iran, after developing the questionnaire based on literature, it was given to 10 experts in health education and promotion. It was revised based on feedbacks from the experts. Then it was given to 20 women to complete. After 10 days, it was given to compete again. The reliability was obtained 0.79 using α of Cronbach and the content validity index was 0.72. 

Phases of this study. This study included three phases of pretest, planning and implementing teaching interventions based on planned behavior theory, and post-test one and two months after the teaching intervention: (i) *Pretest phase.* After obtaining consent from both control and interventions groups, and ensuring confidentiality of information, pretest was completed for both groups. After completing, pretest questionnaires were collected. Then teaching intervention was carried out including four teaching sessions based on planned behavior theory in the intervention group; (ii) *Intervention’s phase.* Teaching sessions were held in four 45 - 60 minute sessions by the investigator in a conference room for the intervention group.^32^^,^^33^ Teaching tools were whiteboard, PowerPoint and pamphlet. For each session, behavioral objectives were identified, and various methods were employed including lecture, group discussion, and personal consultation. First session was held to change attitude in female nurses about osteoporosis, proper assessment of preventive behavior of osteoporosis. Second session was held to increase perceived pressure from the society and relatives about preventive behavior of osteoporosis. Third session was about specific ideas on what a person should perform in preventing osteoporosis. Forth session was about their intent in selecting preventive behavior of osteoporosis.^32^^,^^33^ Before each session, teaching contents of previous session were reviewed. Teaching pamphlet was provided to female nurses. The control group did not receive any teaching: (iii) *Post intervention phase.* Follow up of nurses' behavior was carried out through telephone contact, texting, and consultation for two months after the intervention. Both groups completed the questionnaire of constructs of planned behavior theory one and two months after the teaching intervention. 

Data analysis. Data were analyzed using the SPSS 21. Shapiro-Wilk test was used to examine normality of data. Friedman test was used for comparison of preventive behavior of osteoporosis in the intervention and control groups, and Wilcoxon test was used for the two comparisons. Mann-Whitney U test was used to compare between the control and intervention groups. Significance was considered at 0.05. 

## Results

In this study, 100 female nurses took part in the intervention group (50 subjects) and control groups (50 subjects). Most nurses were under 40 years old in the intervention group (76%) and control group (72%). Results of analysis showed that there were not significant differences between the intervention and control groups in terms of demographic variables (Table 1). 

**Table 1:** Comparison of Demographic variables in intervention and control groups

Variables		**Intervention group (*n*=50)**		Control group (*n*=50)		Statistics χ^2^	***p*-value**
		Frequency	Percent	Frequency	Percent
Age in years	Under 40	38	76	36	72	0.208	0.65
	≥ 40	12	24	14	28		
Marital status	Single	9	18	6	12	0.706	0.40
	married	41	82	44	88		
Education	Bachelor	39	78	42	84	0.585	0.44
	Master	11	22	8	16		
History of osteoporosis	No	35	70	38	76	0.457	0.50
	Yes	15	30	12	24		
Work history in years	0 - 10	17	34	9	18	3.44	0.18
	11 - 20	18	36	24	48		
≥ 21	15	30	17	34

Results of Friedman test showed that means of attitude, abstract norms, behavioral intent, and behavior about preventive behavior of osteoporosis differed significantly one and two months after the teaching intervention compared to before the intervention (*p*<0.05) (Table 2). In the control group, means of attitude, abstract norms, behavioral intent, and behavior about preventive behavior of osteoporosis were not significantly different one and two months after the teaching intervention compared to before the intervention (*p*>0.05). Results of Mann-Whitney U showed that means of attitude, abstract norms, behavioral intent, and behavior were not significantly different between the control and intervention groups before the intervention (*p*>0.05). One and two months after the intervention, there were significantly differences in means of attitude, abstract norms, behavioral intent, and behavior between the control and intervention groups (*p*<0.001). One and two months after the intervention, means of attitude, abstract norms, behavioral intent and behavior were significantly greater in the intervention group compare to the control group.

**Table 2:** Comparison of means of preventive behavior of osteoporosis between the intervention and control groups before, 1 month and 2 months after the intervention

Construct	Time	Intervention Group		Control Group			Statistics		***p*-value** ^*^	
		Mean	SD	Mean	SD
Attitude	Before the intervention	60.12	5.76	54.94	12.46	-1.38	0.166
One month after the intervention	74.98	0.14	53.10	13.55	-5.76	< 0.001
Two months after the intervention	67.10	2.47	54.58	15.55	-4.05	< 0.001
Statistics	87.96		0.484				
*p*-value^**^	< 0.001		0.785				
Abstract Norms	Before the intervention	27.64	4.04	28.96	4.28	-1.75	0.08
	One month after the intervention	39.88	0.33	28.76	8.16	-8.56	< 0.001
	Two months after the intervention	33.78	1.90	28.92	6.98	-3.95	< 0.001
	Statistics	89.55		3.35				
	*p*-value^**^	< 0.001		0.187				
Behavior control	Before the intervention	15.88	4.20	16.68	3.29	-0.942	0.346
	One month after the intervention	15.90	3.89	15.54	3.43	-0.135	0.893
	Two months after the intervention	15.62	4.22	15.60	3.30	-0.031	0.975
	Statistics	89.55		3.82				
	*p*-value^**^	0.053		0.148				
Behavioral Intent	Before the intervention	28.12	4.67	28.04	5.35	-1.23	0.218
	One month after the intervention	39.64	0.75	28.18	4.89	-1.24	0.046
	Two months after the intervention	34.08	1.88	28.38	8.28	2.77	0.006
	Statistics	85.84		2.90				
	*p*-value^**^	< 0.001		0.299				
Behavior	Before the intervention	21.86	2.20	22.54	3.32	-1.58	0.113
	One month after the intervention	32.62	2.95	23.94	3.79	-7.85	< 0.001
	Two months after the intervention	25.90	3.14	22.68	4.07	-4.19	< 0.001
	Statistics	86.58		3.34				
*p*-value^**^	< 0.001		0.188			

*Mann-Whitney U, ** Friedman

In the intervention group, results of Wilcoxon test showed that means of attitude, abstract norms, behavioral intent, and behavior increased significantly one month after the intervention compared to before the intervention and two months after the intervention compared to before the intervention (*p*<0.001). Mean of attitude in nurses decreased significantly two months after the intervention compared to before the intervention (Table 3). 

**Table 3:** Comparison of means constructs of regarding preventive behavior of osteoporosis in the intervention group before, 1 month and 2 months after the intervention

Time Construct		One month after the intervention--Two months after the intervention	Before the intervention-Two months after the intervention	Before the intervention-One month after the intervention
Attitude	Statistics	1.14	-1.86	-0.72
*p*-value *	< 0.001	< 0.001	< 0.001
Abstract Norms	Statistics	1.09	-1.88	-0.790
	*p* -value *	< 0.001	< 0.001	< 0.001
behavioral intent	Statistics	0.690	-1.83	1.14
	*p* -value *	< 0.001	< 0.001	< 0.001
Behavior	Statistics	1.100	-1.84	-0.740
*p* -value *	< 0.001	< 0.001	< 0.001

*Wilcoxon test

A mixed-design ANOVA was conducted to examine the effects of time (Factor) and group on attitude, abstract norms, perceived behavioral control, behavioral intention, and behavior scores. For attitude scores, neither the main effect of time (F (1.86, 182.54) = 2.53, *p*=0.086, partial η² =0.025) nor the interaction between time and group (F (1.86, 182.54) =4.85, *p*=0.010, partial η² =0.047) reached practical significance, indicating a small effect of the intervention on attitudes. Significant main effects of time and significant time × group interactions were observed for abstract norms, perceived behavioral control, behavioral intention, and behavior scores (all *p*<0.05). For abstract norms, large effect sizes were found for both time (partial η² =0.282) and the interaction (partial η² =0.295). Behavioral intention showed moderate effects for time (partial η² =0.116) and the interaction (partial η² =0.126), while perceived behavioral control demonstrated small-to-moderate effects (partial η² =0.054 and 0.0038, respectively).

The strongest effects were observed for behavior scores, with very large effect sizes for time (partial η² =.0498) and the time × group interaction (partial η² =0.365), indicating that the educational intervention produced substantial improvements in behavior over time, with differential effects between groups (Table 4).

**Table 4:** The effects of time (Factor) and group on attitude, abstract norms, perceived behavioral control, behavioral intention, and behavior scores

Origen	Type III sum of squares	Degrees of freedom	Quadratic mean	F	p-value	Partial eta squared
Factor = Attitude Score						
Factor	694.820	1.863	373.037	2.530	0.086	0.025
Factor*Group	1332.847	1.863	715.583	4.852	0.01	0.047
Error (Factor)	26919.000	182.535	147.473	-	-	-
Factor = Abstract Norms Score						
Factor	1677.807	1.671	1003.855	38.421	<0.001	0.282
Factor*Group	1794.607	1.671	1073.738	41.095	<0.001	0.295
Error (Factor)	4279.587	163.794	26.128	-	-	-
Factor = Behavior Control Score						
Factor	25.820	1.650	15.644	5.585	0.007	0.054
Factor*Group	17.780	1.650	10.773	3.846	0.031	0.038
Error (Factor)	453.067	161.742	2.801			
Factor = Behavioral Intent Score						
Factor	576.060	1.259	457.460	12.916	.000	0.116
Factor*Group	631.687	1.259	501.634	14.163	.000	0.126
Error (Factor)	4370.920	123.407	35.419			
Factor = Behavior Score						
Factor	1908.487	1.995	956.577	97.366	.000	0.498
Factor*Group	1105.260	1.995	553.981	56.387	.000	0.365
Error (Factor)	1920.920	195.522	9.825			

## Discussion

This study showed that planned behavior theory affected preventive behaviors of osteoporosis in nurses. According to the results, teaching plan increased significantly attitude in the interventions group. In the control group, mean of attitude about preventive behavior of osteoporosis was not significantly different one and two months after the intervention. Previous studies have shown that intervention plan could improve preventive behavior of osteoporosis in patients; however, less research has been conducted on female nurses. In most studies, positive results have been reported about the effect of teaching interventions based on planned behavior theory. Shakeri Neghad *et al*.^23^ and Pakyar *et al*.^34^ found that teaching intervention based on planned behavior theory affected attitude positively. These results are consistent with our study. In a study by Shah Mohammadi *et al*.,^35^ examined the effect of teaching intervention based on planned behavior theory on preventing osteoporosis in mothers of high school students in Tehran, and Jalili *et al*.^32^ examined the effect of planned behavior theory on preventing osteoporosis in women at healthcare centers. According to the results of these studies, mean of attitude changed significantly after the teaching intervention. 

According to the results of this study, teaching plan increased abstract norms significantly in women in the intervention group. In the control group, mean of abstract norms about preventive behavior of osteoporosis was not significantly different one and two months after the intervention. Consistent with the results of this study, in a study by Jangi *et al*.^35^, lecture and SMS based on planned behavior theory improved diet behavior in preventing osteoporosis in high school students. According to the results of these studies, planning and teaching over 9 months at school showed significant differences two months after the intervention. In another study by Pakyar *et al*.^34^ on middle-aged people, teaching intervention was effective on abstract norms, which is consistent with the results of our study. Shah Mohammadi *et al*.^33^ and Jalili *et al*.^32^ found that mean of abstract norms changed significantly after the intervention. On the other hand, in a study by Jihooni *et al*.,^36^effect of teaching intervention based on planned behavior theory results in improving mean of abstract norms in menopause women. Possible reasons of increasing in the intervention group are attending of physicians, authorities and family members in teaching sessions. 

According to the results of this study, teaching plan did not increase significantly perceived control of behavior in women in the intervention and control groups. Consistent with the results of this study, Jalili *et al*.^32^ examined effect of planned behavior theory on preventing osteoporosis in women at healthcare centers. According to the results of this study, after the intervention mean of perceived control of behavior did not change significantly, which is consistent with the results of this study. Results of this study are contradict with Pakyar *et al*.,^34^ ShahMohammadi *et al*.,^33^ Ates *et al*.^22^ and Şanlıtürk *et al*.^37^ In a study by Pakyar *et al*.^34^ it was found that teaching intervention was effective on perceived control of behavior. In studies by Shah Mohammadi *et al*.^33^ mean of perceived control of behavior changed significantly after teaching intervention. Ates *et al*.^22^ examined behavioral intent for healthy diet in Turkish teachers based on planned behavior theory. They found that mental norm is the most effective factor on behavioral intent for healthy diet. Şanlıtürk *et al*.^37^ examined the effect of a teaching plan based on planned behavior on asthma management and medication compliance. They found that asthma management and medication compliance increased after teaching planned behavior in the intervention group. This difference in results could be related to difference in research population. If people have necessary resources, self-esteem and adequate ability, they perform specific behavior. Limitations like personality, mental status and working condition could affect preventive behavior of osteoporosis.^32^ On the other hand, this difference could be related to limitations of theory including lack of focusing on change behavior, gap of perceived control of behavior and real control of behavior.^38^ According to the results of this study, teaching plan resulted in significant increase in behavioral intent in the intervention group. Results of this study are consistent with results of studies by ShahMohammadi *et al*.^33^, Jalili *et al*.,^32^ Pinar *et al*.,^39^ Pakyar *et al*.^32^ and Andriyaningtiyas *et al*.^40^

According to the results of this study, teaching plan resulted in significant increase in preventive behavior in the intervention group. Results of this study are consistent with results of studies by Jihooni *et al*.^36^ in menopause women, Dehghankar *et al*.^16^ in health volunteers and Abdelrhamn *et al*.^29^ in female teachers. Abdelrhamn *et al*.^29^ found improving in knowledge, performance and planned behavior of in Egyptian female teachers about prevention of osteoporosis. But a study by Ahmadi Tabatabai^41^,teaching interventions based on planned behavior theory was not effective on physical activity, which is not consistent with results of our study. This difference could be related to age of staff 40 - 60 years as age affects type of physical activity. While age of most nurses was under 40 years in our study. 

One of the strengths of this study is that it was the first-time planned behavior theory was conducted on preventive behaviors of osteoporosis in female nurses. In the control group, constructs of preventive behavior of osteoporosis did not change. On the other hand, using theories of behavior increases the effect of health education programs. Given the nature of nursing and its risk, considering osteoporosis as a common disease in female nurses was another strength of this study. There were some limitations in this study. Given self-report of collected data, there was possible inaccuracy in recording data, which was decreased through explaining and ensuring participants about confidentiality of personal information. Another limitation of this study was quasi-experimental design and selecting groups from two hospitals to decrease contamination bias. 

Conclusion. According to the results of this study, planned behavior theory could affect preventive behavior of osteoporosis in female nurses. Results of this study could be employed as a basis for future interventions, particularly in of nurses' role in health education. This study could be used as a framework for designing and conducting teaching interventions for preventing osteoporosis and health promotion in women. 

Ethical considerations. This study was conducted according to the Helsinki declaration 5 and all considerations related to design of this study. This study was approved at the Ethics Committee at Jahrom University of Medical Sciences (IR. JUMS. REC.1402,035). Informed consent was obtained from the participants. Nurses were stated that participation is voluntary. Questionnaires were anonymous, and collected data were confidential. After the intervention, file of teaching plan was provided to the control group. 

## References

[1] Rundasa DT, Ayisa AA, Mekonen EG (2022). Knowledge, health belief, and associated factors towards the prevention of osteoporosis among post-menopausal women in Metu Town, southwest Ethiopia: A community-based cross-sectional study. International Journal of Orthopaedic and Trauma Nursing.

[2] Pezman B, Haghdoost A, Dortaj R (2011). Ultra analysis of prevalence of osteoporosis in Iranian women. A systematic review and meta-analysis. Iranian Journal of Endocrinology and Metabolism.

[3] Gregson CL, Armstrong DJ, Bowden J, Cooper C, Edwards J, Gittoes NJ (2022). UK clinical guideline for the prevention and treatment of osteoporosis. Archives of osteoporosis.

[4] Nouhi E, Esfandiary F, Azizzadeh Forouzi M, dehesh T (2019). Assessing Relationship Osteoporosis Preventive Behaviors and Blood D3 Level among Nurses. Journal of Jiroft University of Medical Sciences.

[5] Chandran M, Brind’Amour K, Fujiwara S, Ha Y-C, Tang H, Hwang J-S (2023). Prevalence of osteoporosis and incidence of related fractures in developed economies in the Asia Pacific region: a systematic review. Osteoporosis International.

[6] Panahi R, Siboni FS, Kheiri M, Ghoozlu KJ, Shafaei M, Dehghankar L (2021). Promoting the adoption of behaviors to prevent osteoporosis using the health belief model integrated with health literacy: quasi-experimental intervention study. BMC Public Health.

[7] Hemmati F, Sarokhani D, Sayehmiri K, Motadayen M (2018). Prevalence of osteoporosis in postmenopausal women in Iran: a systematic review and meta-analysis. The Iranian Journal of Obstetrics, Gynecology and Infertility.

[8] Fahimfar N, Hesari E, Mansourzadeh MJ, Khalagi K, Sanjari M, Hajivalizadeh S (2024). Prevalence of osteoporosis in the Iranian population: a systematic review and meta-analysis. Journal of Diabetes & Metabolic Disorders.

[9] Kelly RR, McDonald LT, Jensen NR, Sidles SJ, LaRue AC (2019). Impacts of psychological stress on osteoporosis: clinical implications and treatment interactions. Frontiers in psychiatry.

[10] Pakyar N, Pashaeypoor S, Poortaghi S, Nezhad AK (2019). Measuring knowledge and prevention behaviors of osteoporosis among middle-aged patients referring to Kermanshah health centers: Application of planned pattern of behavior. Iranian Journal of Nursing Research.

[11] Kackin O, Ciydem E, Aci OS, Kutlu FY (2021). Experiences and psychosocial problems of nurses caring for patients diagnosed with COVID-19 in Turkey: A qualitative study. International Journal of Social Psychiatry.

[12] Khajooee R, Bagherian B, Dehghan M, Azizzadeh Forouzi M (2019). Missed nursing care and its related factors from the points of view of nurses affiliated to Kerman University of Medical Sciences in 2017. Hayat.

[13] Guimarães ALdO, Felli VEA (2016). Notification of health problems among nursing workers in university hospitals. Revista brasileira de Enfermagem.

[14] Bahnam B, Semnani V, Hadadnia F, Mirmohammadkhani M (2016). Vitamin D serum levels in nurses in Semnan educational hospitals and its association with depression. Koomesh.

[15] Feskanich D, Hankinson SE, Schernhammer ES (2009). Nightshift work and fracture risk: the Nurses’ Health Study. Osteoporosis International.

[16] Dehghankar L, Panahi R, Hasannia E, Hemmati F, Siboni FS (2021). The Effect of an Educational Intervention on Health Literacy and the Adoption of Nutritional Preventive Behaviors Related to Osteoporosis Among Iranian Health Volunteers. Journal of Preventive Medicine and Public Health.

[17] Delsouz S, Farrokh Eslamlou H-R, Didarloo A (2021). The Impact of Educational Intervention Based On Health Belief Model On Promoting Health Beliefs Among Male High School Students In Oshnavieh Toward Aids. Nursing And Midwifery Journal.

[18] Sharma M (2021). Theoretical foundations of health education and health promotion.

[19] McKenzie JF, Neiger BL, Thackeray R (2022). Planning, implementing and evaluating health promotion programs.

[20] Ajzen I (1991). The theory of planned behavior. Organizational behavior and human decision processes.

[21] Alami A, Rezaeian-Kochi M-H, Moshki M (2016). Application of theory of planned behavior in predicting intention and action of preventing tobacco use among students of Gonabad University of Medical Sciences. Iranian Journal of Health Education and Health Promotion.

[22] Ates H (2019). Elementary school teachers’ behavioral intentions for healthy nutrition: Extending theory of planned behavior. Health Education.

[23] Shakerinejad Ghodratollah, Baji Zahra, Tehrani Masoumeh, Hajinajaf Saeedeh, Jarvandi Farzaneh (2017). Effectiveness of an Educational Intervention Based on the Theory of Planned Behavior on the Physical Activities of high school female students. Payesh (Health Monitor) Journal.

[24] Rahi S, Ghani M, Alnaser F, Ngah A (2018). Investigating the role of unified theory of acceptance and use of technology (UTAUT) in internet banking adoption context. Management Science Letters.

[25] Jain S, Singhal S, Jain NK, Bhaskar K (2020). Construction and demolition waste recycling: Investigating the role of theory of planned behavior, institutional pressures and environmental consciousness. Journal of Cleaner Production.

[26] Abdolmaleki B, Peyman N, Esmaili H, Tajfard M (2019). Associated factors with the use of health services among postmenopausal women in Mashhad based on the theory of planned behavior: the role of health literacy. Journal of Education and Community Health.

[27] Dilekler İ, Doğulu C, Bozo Ö (2021). A test of theory of planned behavior in type II diabetes adherence: The leading role of perceived behavioral control. Current Psychology.

[28] Yaghobi Z, Hakkak HM, Ghoochani HT, Joveini H, Maheri M, Taherpour M (2019). Factors Affecting the Intention to Choose the Natural vaginal delivery based on the Theory of Planned Behavior among Primigravidae. Journal of Education and Community Health.

[29] Mohamed Abdelrahman B, Abdallah Abdel-Mordy M, Mohamed Saad A (2024). Educational Program Based on Theory of Planned Behavior for Female Teachers regarding Osteoporosis Prevention. Journal of Nursing Science Benha University.

[30] Columb M, Atkinson M (2016). Statistical analysis: sample size and power estimations. BJA Education.

[31] O'Hara J (2008). How I do it: sample size calculations. Clinical Otolaryngoly.

[32] Jalili Z, Hosseini ZS, Shojaei Zade D (2022). The Application of the Theory of Planned Behavior in Preventing Osteoporosis in Women Referring to Comprehensive Health Service Center in Tehran City, 2019-2020. Tolooebehdasht.

[33] Shahmohamadi F, Hoseini M, Matbouei M, Nasiri M (2022). The effect of educational intervention based on the theory of planned behavior aimed at mothers on osteoporosis prevention behaviors in lower secondary school female students. Journal of Education and Health Promotion.

[34] Pakyar N, Poortaghi S, Pashaeypoor S, Sharifi F (2021). Effect of educational program based on theory of planned behavior on osteoporosis preventive behaviors: a randomized clinical trial. BMC Musculoskeletal Disorders.

[35] Jangi M, Esmaeili R, Matlabi M, Saeedi M, Vahedian Shahroodi M (2018). Comparing the effect of lecturing and mobile phone short message service (SMS), based on the theory of planned behavior on improving nutritional behaviors of high school students in the prevention of osteoporosis. International Journal of Pediatrics.

[36] Jeihooni AK, Rakhshani T, Khiyali Z, Ebrahimi MM, Harsini PA (2022). The effect of educational intervention based on theory of planned behavior on behavioral responses of premenopausal women in prevention of osteoporosis. BMC Women's Health.

[37] Şanlıtürk D, Ayaz-Alkaya S (2021). The Effect of a Theory of Planned Behavior Education Program on Asthma Control and Medication Adherence: A Randomized Controlled Trial. The journal of allergy and clinical immunology. In practice.

[38] Saffari M, Shojaeizadeh D (2014). Health education and health promotion.

[39] Pinar G, Pinar T (2020). The Impact of Health Belief Model Based Educational Intervention on Women’s Knowledge, Beliefs, Preventive Behaviors and Clinical Outcomes About Osteoporosis. Sage Open.

[40] Andriyaningtiyas Y, Tamtomo DG, Murti B (2020). Theory of planned behavior and social cognitive theory on the effect of the community health center tertiary preventive behavior among patients with type 2 diabetes mellitus: a multilevel analysis. Journal of Health Promotion and Behavior.

[41] Tabatabaei SVA, Ardabili HE, Haghdoost AA, Dastoorpoor M, Nakhaee N, Shams M (2017). Factors affecting physical activity behavior among women in Kerman based on the theory of planned behavior (TPB). Iranian Red Crescent Medical Journal.

